# Shwannomes rachidiens: étude de séries

**DOI:** 10.11604/pamj.2019.33.199.17921

**Published:** 2019-07-15

**Authors:** Meryem Himmiche, Youssef Joulali, Imane Staouni Benabdallah, Mohammed Benzagmout, Khalid Chakour, Mohammed faiz Chaoui

**Affiliations:** 1 University Hassan II Hospital, Neurosurgery Department, Fez, Morocco

**Keywords:** Shwannome rachidien, IRM médullaire, chirurgie, Spinal shwannoma, medullary MRI, surgery

## Abstract

SLes shwannomes rachidiens sont des tumeurs bénignes qui représentent 30% des tumeurs rachidiennes. Ils se développent à partir des cellules de shwann des racines rachidiennes. Nous rapportons dans cet article l'expérience du service de neurochirurgie du CHU Hassan II de Fès dans la prise en charge des shwannomes rachidiens, du diagnostic aux modalités thérapeutiques sur une période de 13 ans. Les shwannomes rachidiens représentent 19,5% des cas de compression médullaire opéré durant la même période. L'âge moyen était de 45 ans avec une discrète prédominance féminine. La symptomatologie était insidieuse avec un délai de consultation en moyenne de 18 mois, elle est dominée par les douleurs rachidiennes et radiculaires. La moitié de nos patients avaient des déficits neurologiques. L'IRM médullaire a été réalisée chez tous nos patients. La localisation la plus fréquente était au niveau thoracique (40%). Soixante-deux pourcent des shwannomes étaient intra duraux, rarement extra duraux (8%), mixte dans 4%. L'exérèse chirurgicale était complète dans 96% avec recours à une ostéosynthèse dans deux cas et une arthrodèse dans un seul cas. L'étude histologique a confirmé le diagnostic de neurinome bénin dans 23 cas, un cas de shwannome malin et un cas de neurofibrome. L'évolution était favorable chez la majorité des cas, deux cas ont présenté des complications, une infection de la paroi et une aggravation neurologique. L'intérêt de ce travail est d'apprécier les caractéristiques de cette lésion et de comparer les résultats de notre série avec les données de la littérature.

## Abstract (en)

Spinal schwannomas are benign tumors accounting for 30% of all spinal tumors. They originate from the shwann cells of the spinal roots. We report our experience in managing patients with spinal shwannomas, from diagnosis to treatment modalities, in the Department of Neurosurgery at the University Hospital Hassan II in Fez over a period of 13 years. The patients with spinal shwannomas accounted for 19.5% of those with spinal cord compression treated surgically over the same period. The average age of patients was 45 years, with a discreet female predominance. Spinal schwannomas had an insidious onset, then the median of consultation time was 18 months. The main symptoms were spinal and radicular pain. Half of our patients had neurological deficits. Medullary MRI was performed in all of our patients. Spinal schwannomas occurred predominantly in the chest (40%); 62% of shwannomas were intradural lesions, rarely extradural lesions (8%) and mixed lesions(4%). Complete Surgical resection was performed in 96% of cases with osteosynthesis in two cases and arthrodesis in a single case. Histological examination confirmed the diagnosis of benign neurinoma in 23 cases, malignant shwannoma in one case and neurofibroma in one case. Outcome was favorable in the majority of cases, two patients had complications, an infection of the wall and neurological worsening. The interest in the subject of our study is to highlight the features of these lesions and to compare the results of our case series with the data in the literature.

## Introduction

Les schwannomes rachidiens, encore appelés neurinomes, sont des tumeurs souvent bénignes d'évolution lente, développées aux dépens des cellules de Schwann des racines rachidiennes. Décrites initialement en 1910 par Verocay comme une tumeur encapsulée d'un nerf ou d'une racine, à la différence du neurofibrome. Ces tumeurs sont classifiées selon la localisation intra, extra, intra et extra, en sablier, et invasive vis-à-vis de la vertèbre. La symptomatologie est rachidienne et ou radiculaire, d'évolution insidieuse avec dans la plupart de temps un retard diagnostique; ce sont des tumeurs pris à tort pour des pathologies rachidiennes discales et traitées comme telles. L'IRM reste le diagnostic de choix et guide la voie thérapeutique. Le traitement est chirurgical et l'évolution est souvent favorable.

## Méthodes

Nous rapportons dans cet article le résultat d'une étude rétrospective de 25 patients opérés de shwannomes rachidiens au service de neurochirurgie du CHU Hassan II de Fès pour une période de 13 ans. Ce travail a été basé sur l'exploitation de dossiers cliniques, l'interprétation du bilan radiologique, l'analyse des choix thérapeutique et la qualité d'exérèse et l'évolution à court et à long terme.

## Résultats

**Figure 1:**
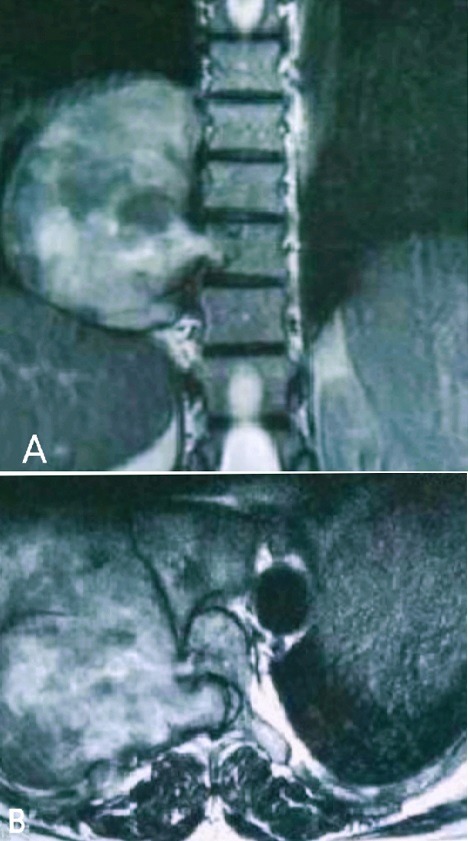
IRM médullaire, séquence T1 avec gadolinium, en coupe coronale (A) et axiale (B) montrant une tumeur en sablier bien limitée, de siège extra dural, de signal hétérogène, siégeant en regard de D7-D8 du côté droit et refoulant la moelle à gauche

**Figure 2:**
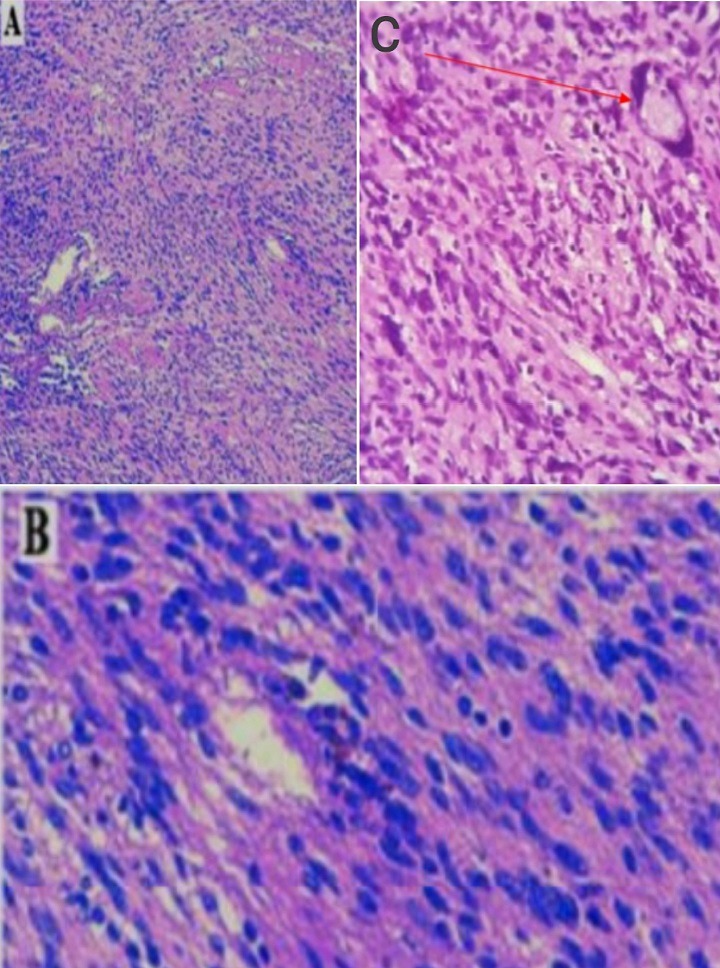
Aspect histologique d'un shwannome bénin (A: HES*50, B: HES*200): prolifération fusocellulaire disposée en nappes; les cellules tumorales sont dotées de noyaux monomorphes allongés avec cytoplasme éosinophile mal limité. Un shwannome malin (C: HES*200): prolifération de cellules atypiques munies des noyaux hyperchromatiques, anisocaryotiques avec des contours irréguliers (flèche)

A la lumière d'une analyse descriptive des différentes données, l'âge des patients a varié entre 9 et 72 ans, avec une moyenne de 44.8 ans avec une prédominance de la tranche d'âge comprise entre 51 et 60 ans. Une prédominance féminine pour un sex ration 14 Femmes/11 Hommes. À noter trois patients ont été suivis pour une hernie discale traitée médicalement. L'évolution des symptômes a varié de 1 mois à 8 ans. La douleur présente le signe fonctionnel le plus retrouvé. La douleur rachidienne a été retrouvée chez 16 patients (64%) répartie surtout au niveau dorso-lombaire. Des névralgies cervico-brachiales dans deux cas des névralgies intercostales dans 4 cas et 3 cas de lombo-cruralgies. Chez 19 patients, la douleur a laissé place au déficit moteur avec une fréquence de 76% dont un cas de tétraparésie. Des troubles sensitifs à type de fourmillement, picotement ont été objectivé chez 5 patients. Les troubles génito-sphinctériens ont été retrouvés chez 11 malades. L'atteinte pyramidale a été présente chez 14 patients, 9 patients avec un Frankel C, 8 patients avec un Frankel D, 6 cas de grade E avec deux cas de paraplégie estimé Frankel B. L'exploration neuroradiologique notamment une IRM médullaire (Figure 1) a été faite chez tous nos patients, objectivant une prédominance de localisation thoracique chez 10 patients, 9 cas de localisation lombaire et 3 cas de localisation cervicale. La charnière cervico-thoracique dans 1 cas, thoraco-lombaire (1 cas) et lombosacré (1 cas). 80% de siège intradural, 12% de siège extradural et 8% de localisation mixte. Cinq patients ont presenté un aspect en sablier très caractéristique des neurinomes rachidiens. Quatre patients ont bénéficié avant leur admission d'une TDM rachidienne, alors qu'une seule patiente en a bénéficié à son admission pour apprécier l'état de la trame osseuse pour une éventuelle fixation post opératoire. La radiographie standard du rachis a été réalisée chez 9 patients objectivant un élargissement du trou de conjugaison dans 3 cas. Les modalités thérapeutiques instaurées à l'admission ont été basées sur un traitement médical à base d'antalgique, corticothérapie avant et après la chirurgie chez les patients déficitaires. Une anticoagulation efficace jusqu'à la reprise de la marche. 23 patients ont été opérés par voie postérieure et deux patients opérés par voie antérieure notamment une thoracotomie. L'exérèse était complète chez 24 patients et un seul cas d'exérèse subtotale sur un volumineux shwannome rachidien lombaire. Deux patients ont bénéficié d'une ostéosynthèse rachidienne pour stabilisation. Ces deux patients ont présenté respectivement un shwannome géant avec une érosion vertébrale de L4 et L5 pour le premier, et une lyse du pédicule osseux de L4 rendue instable pour le second. Un seul patient a bénéficié d'une arthrodèse dorsale par greffon costal. Une radiothérapie a été de mise chez un patient dont l'examen anatomopathologique (Figure 2) a objectivé un shwannome malin ayant reçu 50 Grays en totale. Un cas de neurofibrome a été identifié en étude histologique. L'évolution a été favorable chez 24 patients avec une nette régression de la douleur et absence d'aggravation neurologique. Deux patients ont eu des complications en post opératoire immédiat: un cas d'infection de la cicatrice et un cas d'aggravation neurologique transitoire amélioré avec reprise de son autonomie 5 mois après. L'évolution à long terme a objectivé une nette amélioration clinique chez 22 patients suivis sur une période de 1 an. Alors que deux patients ont été suivis encore plus longtemps, celui porteur d'un shwannome malin et une patiente opérée à deux reprises pour son shwannome de la queue de cheval.

## Discussion

Les schwannomes rachidiens représentent 30% des tumeurs primitives intrarachidiennes. L´incidence des schwannomes rachidiens, selon des études récentes, varie entre 3 et 4 cas / 1 000 000 personnes par an [1]. Ce sont les tumeurs intradurales extra médullaires les plus courantes. . Les schwannomes intramédullaires sont exceptionnels [2]. Les shwannomes rachidiens se présentent souvent sous une forme bénigne unique, la localisation multiple représente 3 à 4% des cas et s'associe souvent avec une neurofibromatose. Pour la plupart des auteurs, l'âge de prédilection se situe entre 30 et 50 ans et le plus souvent lors de la quatrième décade. La tranche d'âge la plus touchée était celle comprise entre 51 et 60 ans, avec une fréquence de 36%. Il paraît alors que la moyenne d'âge de nos patients soit comparable aux autres séries de la littérature. Des résultats disparates dans la littérature pour la répartition selon le sexe. Les neurinomes rachidiens de l'enfant sont rares et représentent 10% des tumeurs rachidiennes [3]. Le schwannome rachidien se caractérise par une latence clinique qui dépend de plusieurs facteurs la localisation et le siège, le caractère évolutif et certains phénomènes d'involution ou de poussée tumorale. La durée d'évolution des symptômes dans notre série était comprise entre 1 mois et 8 ans (96 mois), avec une moyenne de 18 mois. A cet effet, il convient de signaler que le diagnostic est fait tardivement, sources de séquelles invalidantes.

Les signes cliniques les plus révélateurs sont la douleur à la fois rachidienne et radiculaire traduisant le syndrome lésionnel [4]. L'atteinte des voies longues traduit le syndrome sous lésionnel avec des troubles sensitivo-moteurs et troubles sphinctériens. La sémiologie de ses syndromes dépend de la localisation de la lésion et son rapport au cordon médullaire déterminant plusieurs formes cliniques différentes en hauteur et en largeur. D'autre formes cliniques peuvent être révélatrice de cette entité lésionnelle que sont l'hydrocéphalie à pression normal, l'hypertension intracrânienne, la compression médullaire aiguë et parfois un tableau d'hémorragie méningée spinale. Le diagnostic du shwannome rachidien repose sur l'imagerie médullo-rachidienne. L'IRM médullaire est la technique de choix, elle permet une analyse structurale et spatiale de la lésion [5]. Le neurinome apparait en isosignal T1 entouré d'un hyposignal propre au LCR [6], un hypo ou hyper signal T1 est exceptionnel. Il est en hyper signal T2, se rehausse généralement après injection de Gadolinium de façon homogène et intense [7]. La TDM rachidienne apporte des renseignements sur les structures vertébrales et la tumeur se présente sous forme isodense, parfois hétérogène se rehaussant de façon intense. Le myéloscanner constitue l'alternative à l'IRM si contre indiquée. La place des autres explorations radiologiques reste à discuter [8,9]. Les potentiels évoqués somesthésiques peuvent faire partie du bilan préopératoire et renseignent sur l'état fonctionnel de la moelle [10], ils sont parfois utilisés en per opératoire [11].

La chirurgie est le gold standard thérapeutique des shwannomes rachidiens dont l'intérêt est de lever la compression, préserver la continuité anatomique et fonctionnelle du nerf porteur toute en accomplissant une résection chirurgicale complète. Le choix de la voie est orienté par la localisation de la tumeur, le niveau de la lésion et ses rapports intra et extra dural. Parfois un abord combiné est nécessaire pour une meilleure accessibilité de la tumeur [12]. Le recours à une ostéosynthèse pour stabilisation rachidienne est parfois nécessaire. L'intervention est réalisée par microchirurgie dans le but d'une exérèse complète du shwannome toute en préservant la racine porteuse qui est souvent non fonctionnel, mais si elle est viable l'exérèse chirurgicale est parfois incomplète [13]. La radiothérapie a été proposée aux patients qui présentaient des contre-indications opératoires dont le but est de stopper l'évolution tumorale. Parfois la radiothérapie est utilisée en adjuvent à une chirurgie d'exérèse incomplète et ou en cas de shwannomes malin [14]. L'évolution est généralement excellente avec une mortalité presque nulle, plus de 80% de guérison et récupération fonctionnelle. Le risque de récidive dépend étroitement de la qualité d'exérèse [15]. Dans notre série, l'évolution postopératoire immédiate était favorable avec une récupération totale chez 92% des patients. Le pronostic est bon, susceptible d'être influencer par un retard de diagnostic, une exérèse incomplète, un déficit moteur des racines englobées par la tumeur et l'instabilité rachidienne [16].

## Conclusion

Nos résultats en matière de prise en charge chirurgicale des shwannomes rachidiens semblent rejoindre ceux des données de littérature. Le retard diagnostic explique le stade neurologique à l'admission d'où l'intérêt d'un bon examen clinique et neuroradiologique à la moindre suspicion pour un meilleur pronostic.

### Etat des connaissances actuelles sur le sujet

- La suite d'un schwannome est corrélée à l'état neurologique préopératoire du patient;
- La chirurgie des schwannomes rachidiens entraîne généralement de bons résultats fonctionnels post-opératoires.

### Contribution de notre étude à la connaissance

- Rapporter et comparer avec la littérature notre prise en charge clinique et chirurgicale des schwannomes rachidiens;
- Les schwannomes géants et les déficits neurologiques dans notre contexte entraînent des complications post-opératoires et des récidives.

## Conflits des intérêts

Les auteurs ne déclarent aucun conflit d’intérêts.

## References

[1] Emel E, Abdallah A, Sofuoglu OE, Ofluoglu AE, Gunes M, Guler B (2017). Long-term Surgical Outcomes of Spinal Schwannomas: retrospective analysis of 49 consecutive cases. Turk Neurosurg.

[2] Li X, Xu G, Su R, Lv J, Lai X, Yu X (2017). Intramedullary schwannoma of the upper cervical spinal cord: a case study of identification in pathologic autopsy. Forensic Sci Res.

[3] Fischer G, Brotchi J (1994). Intramedullary spinal cord tumors report: French Society of Neurosurgery, 45th annual congress: Angers, June 12/15 1994. Neurochirurgie.

[4] Mireau, Dib Antunes Filho C, Gaudart B (2009). Compression médullaire lente. EM consulte.

[5] Amezyanea T, Pouitb B, Bassouc D, Lecoulesa S, Desraméa J, Bladea JS, Béchadea D, Algayresa JP (2006). Une cause rare de lombosciatique. Elsevier.

[6] Bouaziz M, Derdour S, Laouar O, Lankar A (2012). Spinal root of accessory nerve shwannoma: about a new case. Neurochirurgie.

[7] Afathi M, Peltier E, Adetchessi T, Graillon T, Dufour H, Fuentes S (2015). Minimally invasive transmuscular approach for the treatment of benign intradural extramedullary spinal cord tumours: technical note and results. Neurochirurgie.

[8] Cervoni L, Celli P, Scarpinati M, Cantore G (1994). Neurinomas of the cauda equina clinical analysis of 40 surgical cases. Acta Neurochir (Wien).

[9] Cook AM, Lau TN, Tomlinson MJ, Vaidya M, Wakeley CJ, Goddard P (1998). Magnetic resonance imaging of the whole spine in suspected malignant spinal cord compression: impact on management. Clin Oncol R Coll Radiol G B.

[10] Costa P, Bruno A, Bonzanino M, Massaro F, Caruso L, Vincenzo I (2007). Somatosensory- and motor-evoked potential monitoring during spine and spinal cord surgery. Spinal Cord.

[11] Sala F, Bricolo A, Faccioli F, Lanteri P, Gerosa M (2007). Surgery for intramedullary spinal cord tumors: the role of intraoperative (neurophysiological) monitoring. Eur Spine J.

[12] Banczerowski P, Lipóth L, Vajda J, Veres R (2003). Surgery of ventral intradural midline cervical spinal pathologies via anterior cervical approach: our experience. Ideggyogyaszati Szle.

[13] Madeleine Sowash, Ori Barzilai, Sweena Kahn, Lily McLaughlin, Patrick Boland, Mark Bilsky H, Ilya Laufer (2017). Clinical outcomes following resection of giant spinal schwannomas: a case series of 32 patients. J Neurosurg Spine.

[14] Marchese MJ, McDonald JV (1990). Intramedullary melanotic schwannoma of the cervical spinal cord: report of a case. Surg Neurol.

[15] Ashour A, Rautenberg M, Buhl R, Mehdorn H-M (1999). Giant Ventral Intradural Extramedullary Neuroma. Neurosurgery.

[16] Yamane K, Takigawa T, Tanaka M, Osaki S, Sugimoto Y, Ozaki T (2013). Factors predicting clinical impairment after surgery for cervical spinal schwannoma. Acta Med Okayama.

